# Nursing diagnosis “Inadequate health literacy” in older adults: content validity

**DOI:** 10.1590/0034-7167-2025-0298

**Published:** 2026-03-30

**Authors:** Rachel da Silva Serejo Cardoso, Rosimere Ferreira Santana, Camila Takáo Lopes, Marcos Antônio Gomes Brandão, Priscilla Alfradique de Souza, Tallita Mello Delphino

**Affiliations:** I Universidade La Salle. Niterói, Rio de Janeiro, Brazil; II Universidade Federal Fluminense. Niterói, Rio de Janeiro, Brazil; III Universidade Federal de São Paulo. São Paulo, São Paulo, Brazil; IV Universidade Federal do Rio de Janeiro. Rio de Janeiro, Rio de Janeiro, Brazil; V Universidade Federal do Estado do Rio de Janeiro. Rio de Janeiro, Rio de Janeiro, Brazil; VI Universidade do Estado do Rio de Janeiro. Rio de Janeiro, Rio de Janeiro, Brazil

**Keywords:** Nursing Diagnosis, Health Literacy, Health of the Elderly, Geriatric Nursing, Validation Study., Diagnóstico de Enfermería, Alfabetización en Salud, Salud del Anciano, Enfermería Geriátrica, Estudio de Validación.

## Abstract

**Objectives:**

to assess the content validity of nursing diagnosis “Inadequate health literacy” in older adults.

**Methods:**

a content validity study based on the consensus of 70 professionals in the field who assessed the relevance of each diagnostic component. Components with Content Validity Indexes below 0.80 were modified or reclassified.

**Results:**

all diagnostic definitions achieved satisfactory scores. Related factors with the highest validity index were “deficient social support” and “inadequate/hurried communication from health care professionals”. “Cognitive impairment” was the most prominent associated condition. In the at risk population category, “immigrants and/or non-native language speakers” achieved the highest score. The defining characteristics “difficulty knowing how to act in favor of health” and “lack of information about their health conditions and illness” stood out.

**Conclusions:**

the diagnosis demonstrated satisfactory evidence of content validity, supporting its clinical application.

## RESUMEN

**Objetivos:**

evaluar la validez de contenido del diagnóstico de enfermería “Alfabetización en salud inadecuada” en adultos mayores.

**Métodos:**

este estudio de validación de contenido se basó en el consenso de 70 profesionales del sector, quienes evaluaron la relevancia de cada componente del diagnóstico. Los componentes con índices de validez de contenido inferiores a 0,80 fueron modificados o reclasificados.

**Resultados:**

todas las definiciones diagnósticas obtuvieron puntuaciones satisfactorias. Los factores con mayor índice de validez fueron “apoyo social o apoyo social deficiente” y “comunicación inadecuada/apresurada por parte de los profesionales sanitarios”. El “deterioro cognitivo” fue la afección asociada más prominente. En la categoría de población en riesgo, “inmigrantes y/o hablantes de lenguas no nativas” obtuvieron la puntuación más alta. Las características definitorias “dificultad para saber cómo actuar en favor de la salud” y “falta de información sobre sus afecciones y enfermedades” destacaron.

**Conclusiones:**

el diagnóstico demostró evidencia satisfactoria de validez de contenido, apoyando su aplicación clínica.

## INTRODUCTION

Health literacy is a construct whose discussion began in the 1970s, gaining prominence in recent decades as a social determinant of health and a relevant predictor of clinical outcomes. Its most current definition, proposed by the World Health Organization in 2021, describes it as a set of personal knowledge and skills developed through daily activities, social interactions, and generational experiences. These skills are influenced by organizational structures and available resources, enabling people to access, understand, assess, and apply health information and services to promote and maintain their own health and that of the community^(1)^.

The ability to handle health information is essential for self-care and clinical decision-making, impacting communication with healthcare professionals, adherence to treatment, appropriate use of services, and quality of life^(2)^. Individuals with low health literacy have difficulty understanding health guidelines, which makes them more susceptible to adverse events, such as higher morbidity and mortality, worse clinical indicators, and increased health service costs^(3)^.

In older adults, these effects tend to be even more severe. Studies show that health literacy declines substantially with age, being up to three times lower among older adults compared to younger groups^(4)^. The causes of inadequate health literacy in older adults are multifactorial. Factors such as advanced age, low educational level, limited social support, isolation, low income, multiple chronic diseases, frequent hospitalizations, physical inactivity, and cognitive impairment are strongly associated with reduced health literacy in this population^(5)^. Such conditions make care for this group complex and require a specific diagnostic approach capable of capturing the vulnerabilities generated by the interaction between clinical, functional and social factors that permeate the aging process.

In the context of nursing, the recognition of health literacy as a relevant human response drove its inclusion in NANDA International (NANDA-I) in 2016, with the introduction of diagnosis “Readiness for enhanced health literacy”, in the Health Promotion domain^(6)^. Subsequently, considering the deleterious impacts of low health literacy and the need for autonomous interventions by nurses, diagnosis “Insufficient health literacy” was initially proposed with this name, which was renamed and included in NANDA-I Diagnostic Classification 2024-2026 edition as “Inadequate health literacy”^(7)^.

This is defined as “a pattern of insufficient use and development of a set of skills and competencies (reading proficiency, knowledge, comprehension, motivation, culture and language) to find, understand, assess, and use health concepts and information for daily decision-making, behavior change, health promotion and maintenance, health risk reduction and improvement in overall quality of life”^(7)^.

Initially, this diagnostic proposal for inadequate health literacy was based on the general population and lacked refinement for application to older adults. Therefore, a conceptual analysis was first conducted to identify the attributes, antecedents, and consequences of health literacy in older adults. This allowed us to differentiate the phenomenon from other age groups, supporting the refinement of the diagnostic proposal^(8)^. Subsequently, it was proposed that this content validity be carried out by experts, with a view to ensuring that nursing diagnosis elements (defining characteristics (DCs), related factors (RFs), etc.) are relevant, understandable and comprehensive to identify the health problem in clinical practice of care for older adults.

## OBJECTIVES

To assess evidence of content validity of nursing diagnosis “Inadequate health literacy” in older adults.

## METHODS

### Ethical aspects

The study was conducted in accordance with national and international ethical guidelines and approved by the Research Ethics Committee of the *Universidade Federal Fluminense* School of Medicine, whose opinion is attached to this submission. All participants signed an electronic Informed Consent Form, giving their consent to participate in the study.

### Study design

This is a study of content validity of nursing diagnosis conducted according to recommendations for level of evidence 2.2.2 of NANDA-I Classification, which corresponds to moderate diagnostic content validity^(7)^. The theoretical approach used was the collective wisdom model, according to which the collective assessment of a diverse group can offer more reliable and representative judgments than individual decisions^(9)^. This study followed the Guidelines for Reporting Reliability and Agreement Studies^(10)^ and was conducted entirely remotely from November 2021 to February 2022.

The diagnostic elements assessed came from a previous concept analysis study on insufficient health literacy in older adults^(8)^, including the following diagnostic elements: diagnostic title (DT), definition, RFs, associated conditions (ACs), at risk population (AP), and DCs. The operational and constitutive definitions were constructed based on scientific literature on health literacy in older adults.

### Population, sample, inclusion and exclusion criteria

The proposal by Lopes and Silva^(11)^ was adopted regarding the definition of expertise criteria for collective wisdom and the expanded search for participants who study and/or practice on the object of investigation. The minimum criterion adopted was two years of clinical or academic experience in nursing diagnosis and/or health literacy and/or gerontology, considering that this profile ensures substantive knowledge about the phenomenon investigated and the capacity for informed judgment.

Participants were identified through an active search in the directory of research groups in Brazil (*Lattes* Platform/National Council for Scientific and Technological Development), using the terms “health literacy”, “gerontology”, and “nursing diagnosis” in the title, keywords, or line of research of the groups. Additionally, the snowball sampling technique was used^(11)^, in which the first participants indicated other professionals with the desired profile.

For sample calculation, the formula n₀ = (Z1-α/2 · s / ϭ)^2^, where Z = 1.96 (95% confidence level), s = 0.17 (standard deviation) and ϭ = 0.05 (sampling error)^(12)^ was used, resulting in an estimated minimum of 45 participants. The response period was 15 days, with an extension of 30 days to ensure expert participation.

### Study protocol

Data collection was conducted via an electronic form (Google Forms^®^), sent via an invitation letter to potential participants via email and messaging apps. The collection instrument was divided into two parts. The first contained information about the research, the Informed Consent Form, and participant characteristics (sex, region of practice, academic qualifications, specialization in gerontology, length of professional experience, and clinical or academic experience in nursing diagnosis, health literacy, and/or gerontology).

The second part consisted of an assessment of diagnostic elements (DT, DC, RF, and AC). The relevance of each component was assessed using a 5-point Likert scale, ranging from 1 (completely disagree) to 5 (completely agree), 2 (partially disagree), 3 (indifferent), and 4 (partially agree). Participants were also able to suggest editorial, conceptual, or operational changes in open-ended and free-response fields.

### Analysis of results

The data were organized in Microsoft Office Excel^®^ 2016 spreadsheets and analyzed using the IBM Statistical Package for the Social Sciences version 24. Initially, a descriptive analysis of participants’ characterization variables was performed, with distribution of absolute and relative frequencies.

To analyze the diagnosis content validity, Content Validity Index (CVI) was calculated. After initial data assessments, I-CVI (indicators for each item) and S-CVI/Ave (average variance extracted) indicators were calculated. I-CVI was calculated using the formula: number of responses 4 (partially agree) + 5 (completely agree) divided by the total number of responses^(12)^. Then, S-CVI/Ave was calculated between all items analyzed, corresponding to the sum of all I-CVI divided by the total number of items assessed^(13)^.

The following criteria were adopted for interpreting I-CVI: values ≥ 0.80 were considered as evidence of satisfactory content validity; values between 0.78 and 0.79 were classified as minimum acceptable validity, subject to revision; values below 0.78 indicated the need for modification of an item; values below 0.5 were considered unacceptable. The criterion for content validity based on S-CVI was a value greater than or equal to 0.90^(13)^. Furthermore, Fisher’s exact test was applied to assess whether there was an association between judges’ experience and their responses, with no statistically significant associations being observed (p > 0.05).

Experts’ suggestions, presented in free text in the open field, were analyzed qualitatively. For instance, when suggestions included replacing terms with synonyms to improve clarity and/or measures for accuracy, or when spelling corrections were made, these changes were accepted. Contributions were analyzed by the authors and, when relevant to the focus conceptual domain and NANDA-I criteria, were incorporated into a preliminary version of the diagnostic proposal. This version was submitted to the NANDA-I Diagnosis Development Committee (DDC), which assessed the suggestions received. After this assessment, some diagnostic elements were adjusted in terms of terminology, others were relocated, and some items were removed.

## RESULTS

One hundred experts from various regions of Brazil were invited. Upon acceptance, 75 professionals began completing the instrument. The remaining participants did not respond within the estimated timeframe, without providing a reason for not participating. Of the 75 professionals who began completing the instrument, 70 completed the assessment in full, while five responded incompletely and were excluded.

Females predominated (90%). The majority worked in the Southeast (32.9%) and Midwest (27.1%) regions, followed by the South (20%), Northeast (12.9%), and North (7.1%) regions. Concerning qualifications, 58.6% held a doctoral degree (including completed, in progress, and postdoctoral studies); 27.2% held a master’s degree (completed or in progress); and 14.2% were specialists. The majority (75.7%) did not have a specialization in gerontology, while 21.4% had already completed this training and 2.9% were currently studying. Regarding professional experience, 35.7% of participants had between 10 and 20 years of experience; 25.7% had over 30 years; 18.6% had between 20 and 30 years; and 20% had less than 10 years. Most judges reported experience in nursing diagnoses (84.3%) and gerontology (71.4%), and 35.7% had previous experience related to health literacy.

In this study, all expert assessments regarding the relevance of the proposed diagnostic elements were analyzed, supported by constitutive and operational definitions of the diagnostic elements. These obtained S-CVI ≥ 0.80, thus demonstrating a high degree of agreement among experts.

DT assessment resulted in CVI values of 84.3% and 91.4%, with respective p-values of 0.621 and 0.501. As a suggested conceptual improvement, the term “insufficient” was replaced by “inadequate”, in accordance with NANDA-I terminology standards, considering that “inadequate” represents the absence of quality or necessary criteria, while “insufficient” refers only to quantity. This change was welcomed by the authors and accepted by the NANDA-I DDC, being maintained in the final version of the diagnosis.

As for the diagnostic definition, one of the experts suggested explicitly including older adults in the text, a proposal that was rejected, considering that, although the study focused on this population, the diagnosis should remain generally applicable. Thus, the provisional conceptual wording was maintained: “reduced ability to obtain, process, understand, evaluate, and use/apply health information for daily decision-making for health promotion and maintenance, health risk reduction, and overall improvement in quality of life”^(8)^. Subsequently, after analysis and validity by the DDC, the final version was consolidated and published in NANDA-I Taxonomy (2024-2026) with the title “Inadequate health literacy” and the definition: “Unsatisfactory pattern of obtaining, appraising, and applying basic health information and services needed to make health decisions”^(7)^.

The RFs, presented in Table 1, demonstrated satisfactory validity, with the exception of the item “overwhelmed health care system” (RF4).

**Table 1:** Distribution of expert responses regarding factors related to diagnosis “Inadequate health literacy” in older adults, Niterói, Rio de Janeiro, Brazil, 2025

Code	Related factor	CVI of constitutive definition	CVI of operational definition	*p* value
**RF1**	Deficient social support	0.91	0.91	0.612
**RF2**	Deficient knowledge	0.90	0.80	0.138
**RF3**	Inadequate/hurried communication by health care professionals overestimating understanding skills	0.90	0.91	0.207
**RF4**	Overwhelmed health care system	0.80	0.74	0.088
**RF5**	User’s shame to ask	0.88	0.94	0.366
**RF6**	Health materials produced/written at an educational level above that of the majority of the population without considering the level of health literacy	0.84	0.80	0.451
**S-CVI/Ave**		**0.87**	**0.85**	

*RF - related factor; CVI - Content Validity Index; S-CVI/Ave - averages between the CVIs of the constitutive and operational definition of all related factors assessed.*

*Note: the p values presented refer to the assessment of the operational definition of each item based on Fisher’s exact test.*

RFs are understood as modifiable elements through independent nursing interventions and, therefore, constitute priority targets in the selection of care strategies. In the case of RF4, “overwhelmed health care system”, after expert assessment, was divided into two elements-RF “perceived complexity of health care system” and DC “difficulty navigating complex health care systems”-because it is an observable clinical indicator in this population. These elements are considered particularly relevant in older adults and were included in the final version of the diagnosis.

A similar situation occurred with RF6, “health materials produced/written at an educational level above that of the majority of the population without considering the level of health literacy”, which also underwent terminological adjustments. In the final version of the nursing diagnosis, this factor was broken down into more specific elements, such as “inadequate information available to support person” (specific to older adults) and “perceived complexity of health care information and inadequate information about health care options” (applicable to the general population), enabling better operationalization by nurses.

In the case of RF3, “inadequate/hurried communication by health care professionals overestimating understanding skills”, the need to detail the aspects involved was identified. Thus, this factor was reformulated in more specific and observable terms, such as “inadequate trust in health personnel, dependent on others’ opinions”, “inadequate understanding of information by support person”, and “inadequate communication skills”, with the first three elements highlighted as characteristic of older adults.

RF1, “deficient social support”, was also restructured, resulting in the terms “inadequate social support” and “inadequate social activities” (specific to older adults). Additionally, the factor “inadequate self-efficacy” was included, recognized as a distinct and relevant element in the context of older adults.

Regarding RF5, “user’s shame to ask”, the term was reformulated as “hesitancy to ask questions”, maintaining its specific applicability to older adults. RF2, “deficient knowledge”, was removed from the RF category and, considering its clinical and observable nature, relocated to the set of DCs, which correspond to grouped clinical inferences that express the manifestation of the diagnosis. These results are presented in Table 3 and discussed in the following section.

**Table 2:** Distribution of expert responses regarding associated conditions and at risk populations for diagnosis “Inadequate Health Literacy” among older adults, Niterói, Rio de Janeiro, Brazil, 2025

Code	Element	CVI of constitutive definition	CVI of operational definition	*p* value
**AC1**	Chronic diseases	0.88	0.88	0.930
**AC2**	Sensory deficit	0.94	0.94	0.807
**AC3**	Cognitive impairment	0.97	0.94	0.627
**AC4**	Depressive symptoms	0.92	0.94	0.967
**AC5**	Decline in physical function	0.92	0.92	0.701
**ACS-CVI/Ave**		**0.93**	**0.92**	
**AP1**	Low education	0.88	0.84	0.266
**AP2**	Immigrants and/or not speaking the native language	0.90	0.91	0.708
**AP3**	Economically disadvantaged	0.88	0.94	0.730
**AP4**	Older adult	0.87	0.84	0.995
**AP S-CVI/Ave**		**0.88**	**0.88**	

*AC - associated condition; AP - at risk population; CVI - Content Validity Index; S-CVI/Ave - averages between the CVIs of the constitutive and operational definition of all related factors assessed.*

*Note: the p values presented refer to the assessment of the operational definition of each item based on Fisher’s exact test.*

**Table 3:** Distribution of expert responses regarding the defining characteristics of diagnosis “Inadequate health literacy” in older adults, Niterói, Rio de Janeiro, Brazil, 2025

Code	Defining characteristic	CVI of constitutive definition	CVI of operational definition	*p* value
**DC1**	Difficulty seeking support or health information when needed for health decision-making	0.91	0.90	0.431
**DC2**	Difficulty in knowing how to act in favor of health	0.91	0.91	0.959
**DC3**	Lack of information about their health conditions and illnesses	0.98	0.90	0.143
**DC4**	Difficulty following written or oral information from health care professionals	0.91	0.88	0.972
**DC5**	Difficulty adhering to drug therapy	0.84	0.92	0.480
**DC6**	Worsening health conditions and/or difficulty controlling the disease	0.88	0.90	0.472
**DC7**	Low social participation and less likely to maintain a support network and utilize social resources	0.88	0.91	0.571
**S-CVI/Ave**		**0.90**	**0.90**	

*DC - defining characteristic; CVI - Content Validity Index; S-CVI/Ave - averages between the CVIs of the constitutive and operational definition of all related factors assessed.*

*Note: the p values presented refer to the assessment of the operational definition of each item based on Fisher’s exact test.*

Therefore, the six RFs assessed in this study, after suggested adjustments, resulted in eight specific to older adults: “inadequate social activities”; “inadequate self-efficacy”; “inadequate understanding of information by support person”; “inadequate trust in health personnel”; “dependence on others’ opinions”; “inadequate information available to support person”; “hesitancy to ask questions”; and “perceived complexity of health care information”. These factors more accurately reflect the clinical, functional, and social vulnerabilities of this age group in the context of health literacy.

Table 2 presents the results of content validity of ACs and AP. All components achieved satisfactory validity indices; however, conceptual and terminological adjustments were made that resulted in reclassification, reformulation, or suppression of some elements, with the aim of ensuring greater diagnostic coherence and clinical applicability.

ACs, as defined by NANDA-I, refer to medical diagnoses, diagnostic or therapeutic procedures, and pharmacological preparations that cannot be directly modified by nursing interventions. Based on this criterion, AC4 “depressive symptoms” was relocated to the RF category, as it is considered a condition potentially modifiable by nursing interventions, although not exclusive to older adults. The term “depression”, in turn, was removed from the final version of the diagnosis.

AC2 “sensory deficit”, after assessment by specialists, was divided into two elements - AC “speech disorders” and RF “unaddressed inadequate vision” -, as it is amenable to nursing intervention and is specific to aging. In turn, AC3, “cognitive impairment”, was replaced by “neurocognitive disorders”, adopting a more current and comprehensive terminology for typical conditions present in older adults. Finally, the conditions “chronic diseases” (AC1) and “decline in physical function” (AC5) were reorganized under the terms “chronic diseases”, “acute illness”, and “critical illness”, maintaining their clinical relevance but without age restrictions, as they can affect different population groups.

Among the elements analyzed, the following stand out as specific to older adults, in the set of ACs, as shown in Table 2, “neurocognitive disorders” and “speech disorders”, both maintained in the final version of the diagnosis because they reflect frequent changes in aging that directly impact health literacy. “Polypharmacy”, recognized as a common condition in this age group due to the prevalence of comorbidities and the complexity of treatment regimen, was also incorporated.

In relation to APs, also presented in Table 2, it is noteworthy that AP3, “economically disadvantaged”, was retained in the final version as “economically disadvantaged individuals” because it represents a condition of vulnerability that directly affects health literacy, although not exclusive to aging. AP1, “low education”, and AP2, “immigrants and/or not speaking the native language”, although recognized as relevant by experts during the validity stage, were replaced by the NANDA-I DDC with the more comprehensive term: “socially disadvantaged individuals”. This reformulation allows for the integration of different dimensions of social exclusion under a single category, aligning with the perspective of expanded social risk and increasing the applicability of the diagnosis to different population contexts, without restricting it solely to older adults.

The term “older adult” was excluded within APs, understanding that its inclusion in the category “socially vulnerable individuals” guarantees the representation of this group by encompassing contexts of exclusion and fragility that directly affect the ability to access and use health information.

Table 3 below summarizes the content validity results of the DCs proposed in this study, which represent observable clinical manifestations of diagnosis “Inadequate health literacy” in older adults. The data demonstrate the relevance of the elements assessed, with an S-CVI/Ave of 0.90, indicating a high degree of agreement among experts.

Among the clinical indicators analyzed, various suggestions were adjusted or renamed to better represent observable manifestations in clinical practice and compatible with the context of aging. Some DCs were reformulated based on expert suggestions, such as the transition from “difficulty in knowing how to act in favor of health” to “difficulty with personal health care decision-making” and the expansion of “lack of information about their health conditions and illnesses” to “inadequate knowledge of health care practices”, giving greater scope to the manifestation.

Furthermore, DC “low social participation” was reformulated as “inadequate willingness to participate in social interaction”, recognizing the impact of social isolation on older adults’ engagement in self-care. New DCs were also incorporated into the final version, such as “inadequate understanding of available health care options” and “difficulty navigating complex health care systems”, both derived from specific evidence from this study. These reformulations and inclusions reinforce the role of diagnosis in the early identification of clinical manifestations that affect autonomy, access to information, and decision-making among older adults with inadequate health literacy.

Despite the satisfactory results, after expert assessment, conceptual and terminological adjustments were made, including the reformulation of terms and the inclusion of new elements. These modifications were subsequently reviewed, redefined, and approved by the NANDA-I DDC, which made the necessary refinements, resulting in the final version of the diagnosis published in the 2024-2026 taxonomy^(7)^.

## DISCUSSION

In this study, “Inadequate health literacy” was refined by incorporating elements specific to older adults, validated by experts. The results confirmed that certain RFs, ACs, and DCs reflect vulnerabilities inherent to aging, which led to their inclusion in the final version of the diagnosis published by NANDA-I (2024-2026)^(7)^.

Among the RFs, nine were identified as specific to older adults. “Inadequate social support” stood out with high agreement among experts, as it expresses the fragility of support networks in old age, often impacted by retirement, widowhood, loss of ties, and institutionalization. Although this factor can occur in other age groups, it manifests itself more intensely and frequently among older adults. Recent studies have identified that older adults with low social support are at greater risk of social isolation, which is associated with worse health outcomes, including cognitive impairment, dementia, functional decline, and increased mortality^(14,15)^.

Furthermore, two RFs proposed in the study, “inadequate social activities” and “inadequate self-efficacy”, were validated as specific to older adults and accepted in the final version. Both reflect the effect of inactivity and demotivation on self-care and the active search for health information. Recent studies show that older adults with low social participation have less involvement in preventive practices, poorer health perception, a higher risk of frailty, sleep disturbances, and feelings of loneliness. Similarly, low levels of self-efficacy compromise engagement in caregiving behaviors, hinder decision-making, and are associated with lower adherence to healthy behaviors and a poorer perception of physical and psychological well-being^(16,17)^.

Other RFs also highlight cognitive and communication barriers more prevalent in aging, such as “unaddressed inadequate vision”, “inadequate trust in health personnel”, “hesitancy to ask questions”, “dependence on others’ opinions”, “inadequate information available to support person”, and “inadequate understanding of information by support person”. These factors reflect sensory, emotional, and sociocultural limitations that directly impact the health literacy process in old age, such as difficulty reading labels or instructions, fear of questioning professionals, and delegating decisions to family members or caregivers-conducts that are uncommon in younger adults but common in gerontological and geriatric clinical practice^(18-20)^.

ACs such as “neurocognitive disorders”, “speech disorders”, and “polypharmacy” were retained in the final version of the diagnosis. These conditions, while present at other ages, are notably prevalent and have more serious implications in older adults. “Neurocognitive disorders”, such as mild cognitive impairment and dementia, reduce the ability to understand health advice and perform self-care actions^(21)^. Speech disorders limit the expression of symptoms, doubts or needs, affecting communication with professionals^(22)^. “Polypharmacy”, in turn, is a typical phenomenon of aging, resulting from multimorbidity, and is directly associated with an increased risk of adverse events, medication confusion, and difficulty in therapeutic adherence^(23)^.

The specificity of these ACs in old age lies not only in their prevalence, but in the way they interfere with health literacy, as they hinder assimilation of information, increase dependence on others and require more targeted communication and care strategies^(23)^.

Finally, seven DCs were recognized as specific to older adults in the final version of NANDA-I and were directly derived from this research. They are: “inappropriate seeking of health care services”; “inadequate understanding of available health care options”; “inadequate understanding of health information”; “difficulty implementing a health-related course of action”; “difficulty navigating complex health care systems”; “difficulty with personal health care decision-making”; and “inadequate willingness to participate in social interaction”. These indicators express clinical and behavioral difficulties frequently observed in the care of older adults with health literacy. Older adults with cognitive decline, low education, or little familiarity with the health system often demonstrate difficulty understanding treatment plans, making autonomous decisions, following self-care routines, and utilizing services effectively^(24,25)^.

An “inadequate willingness to participate in social interaction”, for instance, reflects the emotional impact of health literacy, compromising not only engagement in social activities but also the motivation to seek care and follow clinical guidelines, something that has been associated with worsening functionality and psychological well-being. Loneliness is intrinsically linked to human nature and the need for social connection. However, among older adults, especially in vulnerable contexts, this social disconnection is often exacerbated by low levels of health literacy. Studies show that older adults with poor health literacy have fewer resources to understand, access, and utilize health information and services, which contributes to feelings of isolation and chronic loneliness^(26,27)^.

The inclusion of indicators such as “difficulty navigating complex health care systems” also highlights a critical reality in the care of older adults in fragmented systems with inaccessible technical language. Health systems’ poor navigability constitutes a significant barrier to health equity, disproportionately affecting older adults and those with low health literacy^(28)^. Studies have found that older adults with low health literacy face greater barriers to accessing and using health systems effectively, even in contexts with a consolidated healthcare structure^(19)^.

### Study limitations

A limitation of this study is the exclusive participation of Brazilian nurses. However, the comprehensive sample size of the country may be overextended due to the country’s continental nature. Gerontological practices in sociocultural contexts in other countries should be addressed in future clinical studies, given the refinement made in this study, as noted in the recommendations.

### Contributions to nursing, health or public policy

This study offers a significant contribution to gerontology nurses’ clinical practice by proposing a refined diagnostic framework based on elements specific to older adults. The inclusion of RFs, ACs, and clinical indicators representative of the vulnerabilities and particularities of aging strengthens nurses’ ability to identify this human response early and plan more effective interventions. This diagnostic framework contributes to equity in care, autonomy promotion, and health problem prevention. Furthermore, the diagnosis’s acceptance by NANDA-I for inclusion in the 2024-2026 Nursing Diagnosis Classification expands its international dissemination, allowing its translation into more than 20 languages and strengthening its application in different clinical and educational settings.

## CONCLUSIONS

The diagnostic proposal “Inadequate health literacy” has satisfactory evidence of content validity for application to older adults. Expert suggestions were incorporated and refined, resulting in a version conceptually sensitive to the specificities of aging. The use of results presented in clinical validity studies is recommended to test applicability and diagnostic accuracy in care practice contexts. Furthermore, this study contributes to the development of operational definitions that can be used in intervention studies, which is relevant given their growing importance as a determinant of quality of life and well-being for older adults.

## Data Availability

The research data are available within the article.
