# Supplementary material for: Nursing diagnosis “Inadequate health literacy” in older adults: content validity
Source: Rev Bras Enferm. 2026 Mar 30;79:e20250298. doi: 10.1590/0034-7167-2025-0298 (PMC13053012; doi:10.1590/0034-7167-2025-0298)
Supplement: Translation

# Translation: Diagnóstico de enfermagem “Letramento em saúde inadequado” na população idosa: validação de conteúdo

**DOI:** 10.1590/0034-7167-2025-0298pt

**Authors:** Rachel da Silva Serejo Cardoso, Rosimere Ferreira Santana, Camila Takáo Lopes, Marcos Antônio Gomes Brandão, Priscilla Alfradique de Souza, Tallita Mello Delphino

**Affiliations:** I Universidade La Salle. Niterói, Rio de Janeiro, Brasil; II Universidade Federal Fluminense. Niterói, Rio de Janeiro, Brasil; III Universidade Federal de São Paulo. São Paulo, São Paulo, Brasil; IV Universidade Federal do Rio de Janeiro. Rio de Janeiro, Rio de Janeiro, Brasil; V Universidade Federal do Estado do Rio de Janeiro. Rio de Janeiro, Rio de Janeiro, Brasil; VI Universidade do Estado do Rio de Janeiro. Rio de Janeiro, Rio de Janeiro, Brasil

**Keywords:** Diagnóstico de Enfermagem, Letramento em Saúde, Saúde do Idoso, Enfermagem Geriátrica, Estudo de Validação.

## Abstract

**Objetivos:**

avaliar a validade de conteúdo do diagnóstico de enfermagem “Letramento em saúde inadequado” em idosos.

**Métodos:**

estudo de validação de conteúdo baseado no consenso de 70 profissionais da área que avaliaram a relevância de cada componente diagnóstico. Componentes com Índices de Validade de Conteúdo inferiores a 0,80 foram modificados ou reclassificados.

**Resultados:**

todas as definições do diagnóstico atingiram índices satisfatórios. Os fatores relacionados com maior índice de validade foram “apoio social ou suporte social deficiente” e “comunicação inadequada/apressada dos profissionais de saúde”. O “déficit cognitivo” foi a condição associada mais destacada. Na categoria de populações em risco, “imigrantes e/ou não falar a língua nativa” obteve maior índice. As características definidoras “dificuldade em saber agir em prol da saúde” e “déficit de informações sobre suas condições de saúde e doença” destacaram-se.

**Conclusões:**

o diagnóstico demonstrou evidências satisfatórias de validade de conteúdo, apoiando sua aplicação clínica.

## INTRODUÇÃO

O letramento em saúde é um construto sobre o qual a discussão se iniciou na década de 1970, ganhando destaque nas últimas décadas como um determinante social da saúde e um preditor relevante de desfechos clínicos. Sua definição mais atual, proposta pela Organização Mundial da Saúde em 2021, descreve-o como um conjunto de conhecimentos e competências pessoais desenvolvidos por meio de atividades cotidianas, interações sociais e experiências geracionais. Essas competências são influenciadas pelas estruturas organizacionais e recursos disponíveis, permitindo às pessoas acessar, compreender, avaliar, e aplicar informações e serviços de saúde para promoção e manutenção da própria saúde e da coletividade^(1)^.

A capacidade de lidar com informações em saúde é essencial para o autocuidado e a tomada de decisão clínica, impactando a comunicação com profissionais de saúde, a adesão ao tratamento, o uso adequado dos serviços e a qualidade de vida^(2)^. Indivíduos com baixo letramento em saúde apresentam dificuldades em compreender orientações em saúde, o que os torna mais suscetíveis a eventos adversos, como maior morbimortalidade, piores indicadores clínicos e aumento de custos com serviços de saúde^(3)^.

Na população idosa, tais efeitos tendem a ser ainda mais severos. Estudos apontam que o letramento em saúde reduz substancialmente com a idade, sendo até três vezes mais baixo entre idosos em comparação a grupos mais jovens^(4)^. As causas do letramento em saúde inadequado na população idosa são multifatoriais. Fatores como idade avançada, baixo nível educacional, apoio social limitado, isolamento, baixa renda, presença de múltiplas doenças crônicas, hospitalizações frequentes, inatividade física e comprometimento cognitivo estão fortemente associados à redução do letramento em saúde nessa população^(5)^. Tais condições tornam o cuidado a esse grupo complexo e exigem um olhar diagnóstico específico capaz de captar as vulnerabilidades geradas pela interação entre os fatores clínicos, funcionais e sociais que permeiam o processo de envelhecimento.

No âmbito da enfermagem, o reconhecimento do letramento em saúde como resposta humana relevante impulsionou sua inclusão na NANDA Internacional (NANDA-I) em 2016, com a introdução do diagnóstico “Disposição para o letramento em saúde melhorado”, no domínio de Promoção da Saúde^(6)^. Posteriormente, considerando os impactos deletérios do baixo letramento em saúde e a necessidade de intervenções autônomas por parte dos enfermeiros, foi proposto inicialmente o diagnóstico “Letramento em saúde insuficiente” com essa denominação, o qual foi renomeado e incluído na edição 2024-2026 da Classificação de Diagnósticos da NANDA-I como “Letramento em saúde inadequado”^(7)^.

Esse está definido como “um padrão de uso e desenvolvimento insuficiente de um conjunto de habilidades e competências (proficiência em leitura, conhecimento, compreensão, motivação, cultura e linguagem) para encontrar, entender, avaliar e usar conceitos e informações em saúde para a tomada de decisões diária de decisões, mudanças de comportamentos, promoção e manutenção da saúde, redução de riscos à saúde e melhora da qualidade de vida geral”^(7)^.

Inicialmente, tal proposta diagnóstica de letramento em saúde inadequado se encontrava fundamentada na população geral, carecendo de refinamento para aplicação à população idosa. Diante disso, realizou-se, primeiramente, uma análise conceitual para identificar os atributos, antecedentes e consequentes do letramento em saúde na população idosa, o que possibilitou a diferenciação do fenômeno em relação a outras faixas etárias, subsidiando o aprimoramento da proposta diagnóstica^(8)^. Em seguida, propôs-se a realização desta validação de conteúdo por peritos, com vista a garantir que os elementos do diagnóstico de enfermagem (características definidoras (CDs), fatores relacionados (FRs), etc.) sejam relevantes, compreensíveis e abrangentes para identificar o problema de saúde na prática clínica de assistência ao idoso.

## OBJETIVOS

Avaliar evidências de validade de conteúdo do diagnóstico de enfermagem “Letramento em saúde inadequado” para a população idosa.

## MÉTODOS

### Aspectos éticos

O estudo foi conduzido conforme diretrizes éticas nacionais e internacionais, e aprovado pelo Comitê de Ética em Pesquisa da Faculdade de Medicina da Universidade Federal Fluminense, cujo parecer está anexado à presente submissão. Todos os participantes assinaram, eletronicamente, o Termo de Consentimento Livre e Esclarecido, dando consentimento à participação no estudo.

### Desenho do estudo

Estudo de validade de conteúdo de diagnóstico de enfermagem, conduzido conforme recomendações para nível de evidência 2.2.2 da Classificação da NANDA-I, que corresponde à validade moderada de conteúdo diagnóstica^(7)^. A abordagem teórica utilizada foi o modelo da sabedoria coletiva, segundo o qual a avaliação coletiva de um grupo diverso pode oferecer julgamentos mais confiáveis e representativos do que decisões individuais^(9)^. O estudo seguiu as diretrizes do *Guidelines for Reporting Reliability and Agreement Studies* ^(10)^, sendo conduzido integralmente, em formato remoto, no período de novembro de 2021 a fevereiro de 2022.

Os elementos diagnósticos avaliados foram oriundos de estudo prévio de análise de conceito sobre letramento em saúde insuficiente na população idosa^(8)^, incluindo os seguintes elementos diagnósticos: título diagnóstico (TD), definição, FRs, condições associadas (CAs), população em risco (PR) e CDs. As definições operacionais e constitutivas foram construídas com base na literatura científica sobre letramento em saúde na população idosa.

### População, amostra, critérios de inclusão e exclusão

Adotou-se a proposta de Lopes e Silva^(11)^ quanto à definição de critérios de *expertise* da sabedoria coletiva e de busca ampliada de participantes que estudem e/ou pratiquem sobre o objeto da investigação. Adotou-se como critério mínimo possuir dois anos de experiência clínica ou acadêmica em diagnóstico de enfermagem e/ou letramento em saúde e/ou em gerontologia, por considerar que esse perfil assegura conhecimento substantivo sobre o fenômeno investigado e capacidade de julgamento fundamentado.

Os participantes foram identificados por meio de busca ativa no diretório de grupos de pesquisa no Brasil (Plataforma *Lattes*/Conselho Nacional de Desenvolvimento Científico e Tecnológico), utilizando os termos “letramento em saúde”, “gerontologia” e “diagnóstico de enfermagem” no título, palavras-chave ou linha de pesquisa dos grupos. Adicionalmente, foi utilizada a técnica de amostragem por bola de neve^(11)^, na qual os primeiros participantes indicaram outros profissionais com o perfil desejado.

Para o cálculo amostral, utilizou-se a fórmula n₀ = (Z1-α/2 · s / ϭ)^2^, sendo Z = 1,96 (nível de confiança de 95%), s = 0,17 (desvio padrão) e ϭ = 0,05 (erro amostral)^(12)^, resultando no mínimo estimado de 45 participantes. O prazo de resposta foi de 15 dias, com extensão para 30 dias para garantir a adesão de especialistas.

### Protocolo do estudo

A coleta foi realizada via formulário eletrônico (*Google Forms^®^*), enviado por meio de uma carta convite para os potenciais participantes por e-mail e aplicativo de mensagens. O instrumento de coleta foi dividido em duas partes. A primeira continha as informações sobre a pesquisa, o Termo de Consentimento Livre e Esclarecido, e os dados de caracterização dos participantes (sexo, região de atuação, titulação acadêmica, especialização em gerontologia, tempo de atuação profissional, e experiência clínica ou acadêmica em diagnóstico de enfermagem, letramento em saúde e/ou gerontologia).

A segunda parte do instrumento consistiu na avaliação dos elementos diagnósticos (TD, CD, FR e CA). A relevância de cada componente foi julgada utilizando uma escala Likert de 5 pontos, variando de 1 (discordo totalmente) a 5 (concordo totalmente), passando por 2 (discordo parcialmente), 3 (indiferente) e 4 (concordo parcialmente). Além disso, os participantes puderam sugerir alterações redacionais, conceituais ou operacionais em campos de resposta aberta e livre.

### Análise dos resultados

Os dados foram organizados em planilhas do *Microsoft Office Excel^®^ 2016* e analisados por meio do *software* IBM *Statistical Package for the Social Sciences*, versão 24. Inicialmente, realizou-se análise descritiva das variáveis de caracterização dos participantes, com distribuição de frequências absolutas e relativas.

Para análise da validade de conteúdo do diagnóstico, calculou-se o Índice de Validade de Conteúdo (IVC). Após avaliações iniciais dos dados, calcularam-se os indicadores de I-IVC (indicadores de cada item) e S-IVC/Ave (média dos IVCs para todos os índices da escala/*Average variance extracted*). O I-IVC foi calculado pela fórmula: número de respostas 4 (concordo parcialmente) + 5 (concordo totalmente) dividido pelo total de respostas^(12)^. Em seguida, foi calculado o S-IVC/Ave entre todos os itens analisados, correspondendo à soma de todos os I-IVC dividido pelo total de itens avaliados^(13)^.

Foram adotados os seguintes critérios para interpretação do I-IVC: valores ≥ 0,80 foram considerados como evidência de validade de conteúdo satisfatória; valores entre 0,78 e 0,79 foram classificados como validade mínima aceitável, passíveis de revisão; valores inferiores a 0,78 indicaram necessidade de modificação do item; valores inferiores a 0,5 foram considerados inaceitáveis. O critério para validade de conteúdo a partir do S-IVC foi o valor maior ou igual a 0,90^(13)^. Além disso, foi aplicado o teste exato de Fisher para avaliar se havia associação entre a experiência dos juízes e suas respostas, não sendo observadas associações estatisticamente significativas (p > 0,05).

As sugestões dos especialistas realizadas em texto livre em campo aberto foram analisadas qualitativamente. Como exemplo, quando eram sugeridas substituições de termos por sinônimos para melhorar a clareza e/ou de medidas para precisão, ou quando eram realizadas correções de grafia, tais modificações foram acatadas. As contribuições foram analisadas pelos autores e, quando pertinentes ao domínio conceitual do foco e aos critérios da NANDA-I, foram incorporadas em uma versão preliminar da proposta diagnóstica. Essa versão foi submetida ao *Diagnosis Development Committee* (DDC) da NANDA-I, que avaliou as sugestões recebidas. Após essa avaliação, alguns elementos diagnósticos foram ajustados quanto à terminologia, outros foram realocados, e alguns itens foram suprimidos.

## RESULTADOS

Foram convidados 100 especialistas de diversas regiões do Brasil. Após aceite, 75 profissionais iniciaram o preenchimento do instrumento. Os demais convidados não responderam ao convite no tempo estimado para resposta, sem explicitar o motivo de não participar. Entre os 75 profissionais que iniciaram o preenchimento do instrumento, 70 completaram integralmente a avaliação, e cinco responderam de modo incompleto e foram excluídos.

Houve predominância do sexo feminino (90%). A maioria atuava nas regiões Sudeste (32,9%) e Centro-Oeste (27,1%), seguidas das regiões Sul (20%), Nordeste (12,9%) e Norte (7,1%). Quanto à titulação, 58,6% possuíam formação em nível de doutorado (considerando doutorado concluído, em andamento e pós-doutorado); 27,2% tinham mestrado (concluído ou em andamento); e 14,2% eram especialistas. A maioria (75,7%) não possuía especialização em gerontologia, enquanto 21,4% já haviam concluído essa formação e 2,9% estavam cursando. Em relação ao tempo de experiência profissional, 35,7% dos participantes tinham entre 10 e 20 anos de atuação; 25,7% possuíam mais de 30 anos; 18,6% possuíam entre 20 e 30 anos; e 20% tinham menos de 10 anos. A maior parte dos juízes relatou experiência em diagnósticos de enfermagem (84,3%) e gerontologia (71,4%), e 35,7% tinham atuação prévia relacionada ao letramento em saúde.

Neste estudo, foram analisadas todas as avaliações dos especialistas quanto à relevância dos elementos diagnósticos propostos, apoiados por definições constitutivas e operacionais dos elementos diagnósticos. Essas obtiveram S-IVC ≥ 0,80, evidenciando-se, assim, alto grau de concordância entre os especialistas.

A avaliação do TD resultou em valores de IVC de 84,3% e 91,4%, com respectivos valores de p de 0,621 e 0,501. Como sugestão de melhoria conceitual, foi proposta a substituição do termo “insuficiente” por “inadequado”, em conformidade com os padrões terminológicos da NANDA-I, considerando que “inadequado” representa ausência de qualidade ou critério necessário, enquanto “insuficiente” refere-se apenas à quantidade. A alteração foi acolhida pelos autores e aceita pelo DDC da NANDA-I, sendo mantida na versão final do diagnóstico.

Com relação à definição diagnóstica, um dos especialistas sugeriu a inclusão explícita da população idosa no texto, proposta que não foi acatada, considerando que, embora o estudo tenha sido conduzido com foco nessa população, o diagnóstico deve manter-se aplicável de forma geral. Assim, foi mantida a redação conceitual provisória: “capacidade reduzida de obter, processar, compreender, avaliar e usar/aplicar informações de saúde para a tomada de decisões diárias para promoção e manutenção da saúde, redução de riscos à saúde e melhoria geral da qualidade de vida”^(8)^. Posteriormente, após análise e validação pelo DDC, foi consolidada e publicada a versão final do diagnóstico na Taxonomia NANDA-I (2024-2026) com o título “Letramento em saúde inadequado” e a definição: “Padrão insatisfatório de obtenção, avaliação e aplicação de informações e serviços de saúde necessários à tomada de decisão em saúde”^(7)^.

Os FRs, apresentados na Tabela 1, demonstraram validade satisfatória, com exceção do item *“*Sistema de saúde sobrecarregado” (FR4).

**Tabela 1:** Distribuição das respostas dos especialistas em relação aos fatores relacionados do diagnóstico “Letramento em saúde inadequado” na população idosa, Niterói, Rio de Janeiro, Brasil, 2025

Código	Fator relacionado	IVC da definição constitutiva	IVC da definição operacional	Valor de *p*
**FR1**	Apoio social ou suporte social deficiente	0,91	0,91	0,612
**FR2**	Conhecimento deficiente	0,90	0,80	0,138
**FR3**	Comunicação inadequada/apressada dos profissionais de saúde superestimando habilidades de entendimento	0,90	0,91	0,207
**FR4**	Sistema de saúde sobrecarregado	0,80	0,74	0,088
**FR5**	Vergonha do usuário em perguntar	0,88	0,94	0,366
**FR6**	Materiais de saúde produzidos/escritos no nível educacional acima da maioria da população sem considerar o nível de letramento em saúde	0,84	0,80	0,451
**S-IVC/Ave**		**0,87**	**0,85**	

*FR - fator relacionado; IVC - Índice de Validade de Conteúdo; S-IVC/Ave - médias entre os IVCs da definição constitutiva e operacional de todos os fatores relacionados avaliados.*

*Nota: os valores de p apresentados se referem à avaliação da definição operacional de cada item com base no teste exato de Fisher.*

Os FRs são compreendidos como elementos modificáveis por meio de intervenções de enfermagem independentes e, portanto, constituem alvos prioritários na seleção de estratégias de cuidado. No caso do FR4, “sistema de saúde sobrecarregado”, após avaliação dos peritos, foi desdobrado em dois elementos - FR “percepção de complexidade do sistema de saúde” e CD “dificuldade em navegar por sistemas de saúde complexos” -, por ser um indicador clínico observável nessa população. Tais elementos são considerados particularmente relevantes na população idosa, sendo incluídos na versão final do diagnóstico.

Situação semelhante ocorreu com o FR6 “materiais de saúde produzidos/escritos no nível educacional acima da maioria da população sem considerar o nível de letramento em saúde”, que também passou por ajustes terminológicos. Na versão final do diagnóstico de enfermagem, este fator foi desmembrado em elementos mais específicos, como “informações inadequadas disponíveis à pessoa de apoio” (específico da população idosa) e “percepção de complexidade das informações sobre cuidados à saúde e informações inadequadas sobre opções de cuidados de saúde” (aplicáveis à população geral), possibilitando melhor operacionalização por parte dos enfermeiros.

No caso do FR3, “comunicação inadequada/apressada dos profissionais de saúde superestimando habilidades de entendimento”, identificou-se a necessidade de detalhamento dos aspectos envolvidos. Assim, reformulou-se esse fator em termos mais específicos e observáveis, como “confiança inadequada na equipe de saúde, dependente da opinião dos outros”, “compreensão inadequada de informações pela pessoa de apoio” e “habilidade de comunicação inadequada”, sendo os três primeiros elementos destacados como característicos da população idosa.

O FR1, “apoio social ou suporte social deficiente”, também foi reestruturado, originando os termos “apoio social inadequado” e “atividades sociais inadequadas” (específico da população idosa). Adicionalmente, foi incluído o fator “autoeficácia inadequada”, reconhecido como um elemento distinto e relevante no contexto da pessoa idosa.

No que se refere ao FR5, “vergonha do usuário em perguntar”, o termo foi reformulado como “hesitação em fazer perguntas”, mantendo sua aplicabilidade específica à população idosa. Já o FR2 “conhecimento deficiente” foi suprimido da categoria de FR, e considerando sua natureza clínica e observável, realocado para o conjunto de CDs, as quais correspondem a inferências clínicas agrupadas que expressam a manifestação do diagnóstico. Esses resultados serão apresentados na Tabela 3 e discutidos na seção seguinte.

**Tabela 2:** Distribuição das respostas dos especialistas em relação às condições associadas e às populações em risco do diagnóstico “Letramento em saúde inadequado” na população idosa, Niterói, Rio de Janeiro, Brasil, 2025

Código	Elemento	IVC da definição constitutiva	IVC da definição operacional	Valor de *p*
**CA1**	Doença crônica	0,88	0,88	0,930
**CA2**	Déficit sensorial	0,94	0,94	0,807
**CA3**	Déficit cognitivo	0,97	0,94	0,627
**CA4**	Sintomas depressivos/depressão	0,92	0,94	0,967
**CA5**	Declínio da função física	0,92	0,92	0,701
**S-IVC/Ave CA**		**0,93**	**0,92**	
**PR1**	Baixa escolaridade	0,88	0,84	0,266
**PR2**	Imigrantes e/ou não falar a língua nativa	0,90	0,91	0,708
**PR3**	Desfavorecido economicamente	0,88	0,94	0,730
**PR4**	Idoso	0,87	0,84	0,995
**S-IVC/Ave PR**		**0,88**	**0,88**	

*CA - condição associada; PR - população em risco; IVC - Índice de Validade de Conteúdo; S-IVC/Ave - médias entre os IVCs da definição constitutiva e operacional de todos os fatores relacionados avaliados.*

*Nota: os valores de p apresentados se referem à avaliação da definição operacional de cada item com base no teste exato de Fisher.*

**Tabela 3:** Distribuição das respostas dos especialistas em relação às características definidoras do diagnóstico “Letramento em saúde inadequado” na população idosa, Niterói, Rio de Janeiro, Brasil, 2025

Código	Característica definidora	IVC da definição constitutiva	IVC da definição operacional	Valor de *p*
**CD1**	Dificuldade em buscar apoio ou informações de saúde quando necessárias para tomada de decisão em saúde	0,91	0,90	0,431
**CD2**	Dificuldade em saber agir em prol da saúde	0,91	0,91	0,959
**CD3**	Déficit de informações sobre suas condições de saúde e doença	0,98	0,90	0,143
**CD4**	Dificuldade de seguir informações escritas ou orais de profissionais de saúde	0,91	0,88	0,972
**CD5**	Dificuldade de adesão à terapia medicamentosa	0,84	0,92	0,480
**CD6**	Agravamento das condições de saúde e/ou dificuldade de controlar a doença	0,88	0,90	0,472
**CD7**	Baixa participação social e menos propensos a manter uma rede de apoio e a utilizar recursos sociais	0,88	0,91	0,571
**S-IVC/Ave**		**0,90**	**0,90**	

*CD - característica definidora; IVC - Índice de Validade de Conteúdo; S-IVC/Ave - médias entre os IVCs da definição constitutiva e operacional de todos os fatores relacionados avaliados.*

*Nota: os valores de p apresentados se referem à avaliação da definição operacional de cada item com base no teste exato de Fisher.*

Sendo assim, os seis FRs avaliados neste estudo, após ajustes sugeridos, resultaram em oito específicos da população idosa, a saber: “atividades sociais inadequadas”; “autoeficácia inadequada”; “compreensão inadequada de informações pela pessoa de apoio”; “confiança inadequada na equipe de saúde”; “dependente da opinião dos outros”; “informações inadequadas disponíveis à pessoa de apoio”; “hesitação em fazer perguntas”; “percepção de complexidade das informações sobre cuidados à saúde”. Tais fatores refletem com maior precisão as vulnerabilidades clínicas, funcionais e sociais deste grupo etário no contexto do letramento em saúde.

Na Tabela 2, apresentam-se os resultados da validade de conteúdo das CAs e PR. Todos os componentes alcançaram índices de validade satisfatórios, porém foram realizados ajustes conceituais e terminológicos que resultaram na reclassificação, reformulação ou supressão de alguns elementos, com o objetivo de garantir maior coerência diagnóstica e aplicabilidade clínica.

As CAs, conforme a definição da NANDA-I, referem-se a diagnósticos médicos, procedimentos diagnósticos ou terapêuticos e preparações farmacológicas que não podem ser modificadas diretamente por intervenções de enfermagem. Com base nesse critério, a CA4 “sintomas depressivos” foi realocada para a categoria de FR, por ser considerada uma condição potencialmente modificável por intervenções de enfermagem, embora não exclusiva da população idosa. O termo “depressão”, por sua vez, foi suprimido da versão final do diagnóstico.

A CA2 “déficit sensorial”, após a avaliação dos especialistas, foi desdobrada em dois elementos - CA “distúrbios da fala” e FR “visão inadequada não abordada” -, por ser passível de intervenção de enfermagem e específica do envelhecimento. Por sua vez, a CA3 “déficit cognitivo” foi substituída por “transtornos neurocognitivos”, adotando uma terminologia mais atual e abrangente para condições típicas presente nas pessoas idosas. Por fim, as condições “doença crônica” (CA1) e “declínio da função física” (CA5) foram reorganizadas sob os termos “doença crônica”, “doença aguda” e “doença crítica”, mantendo sua relevância clínica, mas sem restrição etária, visto que podem afetar diferentes grupos populacionais.

Entre os elementos analisados, destacam-se como específicos da população idosa, no conjunto das CAs, conforme a Tabela 2, os “transtornos neurocognitivos” e “distúrbios da fala”, ambos mantidos na versão final do diagnóstico por refletirem alterações frequentes no envelhecimento que impactam diretamente o letramento em saúde. Foi também incorporada a condição “polifarmácia”, reconhecida como uma CA presente nessa faixa etária devido à prevalência de comorbidades e à complexidade do regime terapêutico.

No que se refere às PRs, também apresentadas na Tabela 2, destaca-se que a PR3 “desfavorecido economicamente” foi mantida na versão final como “indivíduos desfavorecidos economicamente” por representar uma condição de vulnerabilidade que afeta diretamente o letramento em saúde, embora não seja exclusiva do envelhecimento. Já PR1 “baixa escolaridade” e PR2 “imigrantes e/ou não falar a língua nativa”, apesar de reconhecidas como relevantes pelos especialistas durante a etapa de validação, foram substituídas pelo DDC da NANDA-I pelo termo mais abrangente: “indivíduos desfavorecidos socialmente”. Essa reformulação permite integrar diferentes dimensões de exclusão social sob uma única categoria, alinhando-se à perspectiva de risco social ampliado e aumentando a aplicabilidade do diagnóstico para diferentes contextos populacionais, sem restringi-lo apenas à pessoa idosa.

No âmbito das PRs, o termo “idoso” foi excluído, entendendo-se que sua inclusão na categoria de “indivíduos socialmente vulneráveis” garante a representatividade desse grupo, ao abarcar contextos de exclusão e fragilidade que afetam diretamente a capacidade de acesso e utilização da informação em saúde.

A seguir, apresenta-se a Tabela 3, que reúne os resultados da validade de conteúdo das CDs propostas neste estudo, as quais representam manifestações clínicas observáveis do diagnóstico “Letramento em saúde inadequado” na população idosa. Os dados evidenciam a relevância dos elementos avaliados com um S-IVC/Ave de 0,90, indicando alto grau de concordância entre os especialistas.

Entre os indicadores clínicos analisados, distintas sugestões foram ajustadas ou renomeadas para melhor representar manifestações observáveis na prática clínica e compatíveis com o contexto do envelhecimento. Algumas CDs foram reformuladas com base nas sugestões dos especialistas, como a transição de “dificuldade em saber agir em prol da saúde” para “dificuldade em tomar decisões pessoais sobre cuidados com a saúde” e a ampliação de “déficit de informações sobre suas condições de saúde e doença” para “conhecimento inadequado das práticas de assistência à saúde”, conferindo maior abrangência à manifestação.

Além disso, a CD “baixa participação social” foi reformulada como “vontade diminuída de participar em interação social”, reconhecendo o impacto do isolamento social sobre o engajamento do idoso no autocuidado. Também foram incorporadas novas CDs à versão final, como “compreensão inadequada das opções de cuidados à saúde disponíveis” e “dificuldade em navegar por sistemas de saúde complexos”, ambas derivadas das evidências específicas do presente estudo. Essas reformulações e inclusões reforçam o papel do diagnóstico na identificação precoce de manifestações clínicas que afetam a autonomia, o acesso à informação e a tomada de decisão entre pessoas idosas com letramento em saúde inadequado.

Apesar dos resultados satisfatórios apresentados, após a avaliação dos especialistas, foram realizados ajustes conceituais e terminológicos, incluindo a reformulação de termos e a inclusão de novos elementos. Essas modificações foram posteriormente analisadas, redefinidas e aprovadas pelo DDC da NANDA-I, que promoveu os devidos refinamentos, resultando na versão final do diagnóstico publicada na taxonomia 2024-2026^(7)^.

## DISCUSSÃO

Neste estudo, o diagnóstico de enfermagem “Letramento em saúde inadequado” foi refinado por meio da incorporação de elementos específicos à população idosa, validados por especialistas. Os resultados confirmaram que determinados FRs, CAs e CDs refletem vulnerabilidades próprias do envelhecimento, o que motivou sua inclusão na versão final do diagnóstico publicada pela NANDA-I (2024-2026)^(7)^.

Entre os FRs, nove foram identificados como específicos da população idosa. O “apoio social inadequado” destacou-se com alta concordância entre os especialistas, por expressar a fragilidade das redes de suporte na velhice, frequentemente impactadas por aposentadoria, viuvez, perda de vínculos e institucionalização. Embora esse fator possa ocorrer em outras faixas etárias, ele se manifesta de forma mais intensa e frequente entre os idosos. Estudos recentes identificou que idosos com baixo apoio social apresentam maior risco de isolamento social, o que está associado a piores desfechos em saúde, incluindo comprometimento cognitivo, demência, declínio funcional e aumento da mortalidade^(14,15)^.

Além disso, dois FRs propostos no estudo, como “atividades sociais inadequadas” e “autoeficácia inadequada”, foram validados como específicos da pessoa idosa e aceitos na versão final. Ambos refletem o efeito da inatividade e da desmotivação sobre o autocuidado e a busca ativa por informações de saúde. Estudos recentes demonstram que idosos com baixa participação social apresentam menor envolvimento com práticas preventivas, pior percepção de saúde, maior risco de fragilidade, distúrbios do sono e sentimentos de solidão. Da mesma forma, níveis reduzidos de autoeficácia comprometem o engajamento em condutas de cuidado, dificultam a tomada de decisão, e estão associados à menor adesão a comportamentos saudáveis e à pior percepção de bem-estar físico e psicológico^(16,17)^.

Outros FRs também evidenciam barreiras cognitivas e comunicacionais mais prevalentes no envelhecimento, como “visão inadequada não abordada”, “confiança inadequada na equipe de saúde”, “hesitação em fazer perguntas”, “dependente da opinião dos outros”, “informações inadequadas disponíveis à pessoa de apoio” e “compreensão inadequada de informações pela pessoa de apoio”. Tais fatores refletem limitações sensoriais, emocionais e socioculturais que impactam diretamente o processo de letramento em saúde na velhice, como dificuldade para ler rótulos ou instruções, receio de questionar profissionais e delegação das decisões a familiares ou cuidadores condutas pouco frequentes em adultos mais jovens, mas comuns na prática clínica gerontológica e geriátrica^(18-20)^.

Entre as CAs, foram mantidas na versão final do diagnóstico as expressões “transtornos neurocognitivos”, “distúrbios da fala” e “polifarmácia”. Essas condições, embora possam estar presentes em outras idades, são notadamente prevalentes e com implicações mais graves na população idosa. Os “transtornos neurocognitivos”, como o comprometimento cognitivo leve e as demências, reduzem a capacidade de compreender orientações de saúde e executar ações de autocuidado^(21)^. Já os “distúrbios da fala” limitam a expressão de sintomas, dúvidas ou necessidades, afetando a comunicação com os profissionais^(22)^. A “polifarmácia”, por sua vez, é um fenômeno típico do envelhecimento, resultante da multimorbidade, e está diretamente associada ao aumento do risco de eventos adversos, confusão medicamentosa e dificuldade no seguimento terapêutico^(23)^.

A especificidade dessas CAs na velhice não reside apenas na sua prevalência, mas na forma como interferem no letramento em saúde, pois dificultam a assimilação de informações, aumentam a dependência de terceiros e exigem estratégias de comunicação e cuidado mais direcionadas^(23)^.

Por fim, sete CDs foram reconhecidas como específicas da população idosa na versão final da NANDA-I e tiveram origem direta na presente pesquisa. São elas: “busca inapropriada de serviços de saúde”; “compreensão inadequada das opções de cuidados à saúde disponíveis”; “compreensão inadequada de informações de saúde”; “dificuldade em implementar um curso de ação relacionado à saúde”; “dificuldade em navegar por sistemas de saúde complexos”; “dificuldade em tomar decisões pessoais sobre cuidados com a saúde”; “vontade diminuída de participar em interação social”. Esses indicadores expressam dificuldades clínicas e comportamentais observadas com frequência no cuidado à pessoa idosa com letramento em saúde. Idosos com declínio cognitivo, baixa escolaridade ou pouca familiaridade com o sistema de saúde frequentemente demonstram dificuldade para entender planos terapêuticos, tomar decisões autônomas, seguir rotinas de autocuidado e utilizar os serviços de forma eficaz^(24,25)^.

A “vontade diminuída de participar em interação social”, por exemplo, reflete o impacto emocional do letramento em saúde, comprometendo não apenas o engajamento em atividades sociais, mas também a motivação para buscar cuidados e seguir orientações clínicas, algo que tem sido associado à piora da funcionalidade e do bem-estar psicológico. A solidão está intrinsecamente ligada à natureza humana e à necessidade de conexão social. No entanto, entre a população idosa, especialmente em contextos de vulnerabilidade, essa desconexão social é frequentemente agravada por baixos níveis de letramento em saúde. Estudos demonstram que idosos com letramento em saúde têm menos recursos para compreender, acessar, e utilizar informações e serviços de saúde, o que contribui para sentimentos de isolamento e solidão crônica^(26,27)^.

A inclusão de indicadores como “dificuldade em navegar por sistemas de saúde complexos” também evidencia uma realidade crítica no atendimento às pessoas idosas em sistemas fragmentados e com linguagem técnica pouco acessível. A baixa navegabilidade dos sistemas de saúde constitui uma barreira importante à equidade em saúde, afetando de forma desproporcional os idosos e aqueles com baixo letramento em saúde^(28)^. Estudos observaram que idosos com baixo letramento em saúde enfrentam maiores barreiras para acessar e utilizar os sistemas de saúde de forma eficaz, mesmo em contextos com estrutura assistencial consolidada^(19)^.

### Limitações do estudo

Uma limitação do estudo se refere à participação exclusiva de enfermeiros brasileiros, no entanto a amostra abrangente ao território pode extrapolar devido ao aspecto continental do País. Considera-se que as práticas gerontológicas em contextos socioculturais de outros países devem ser abordadas em futuros estudos clínicos, devido ao refinamento realizado neste estudo, como apontado nas recomendações.

### Contribuições para a área da enfermagem, saúde ou políticas públicas

Este estudo oferece uma contribuição significativa para a prática clínica do enfermeiro em gerontologia, ao propor uma estrutura diagnóstica refinada com base em elementos específicos da população idosa. A inclusão de FRs, CAs e indicadores clínicos representativos das vulnerabilidades e particularidades do envelhecimento fortalece a capacidade do enfermeiro em identificar precocemente essa resposta humana e planejar intervenções mais eficazes. Tal estrutura diagnóstica contribui para a equidade no cuidado, a promoção da autonomia e a prevenção de agravos em saúde. Além disso, a aceitação do diagnóstico pela NANDA-I para inclusão na Classificação de Diagnósticos de Enfermagem 2024-2026 amplia sua disseminação internacional, permitindo sua tradução em mais de 20 idiomas e fortalecendo sua aplicação em diferentes cenários clínicos e educacionais.

## CONCLUSÕES

A proposta diagnóstica “Letramento em saúde inadequado” reúne satisfatórias evidências de validade de conteúdo para aplicação na população idosa. As sugestões dos especialistas foram incorporadas e aprimoradas, resultando em uma versão conceitualmente sensível às especificidades do envelhecimento. Recomenda-se o uso dos resultados apresentados em estudos de validação clínica para testar a aplicabilidade e a precisão diagnóstica em contextos de práticas de cuidado. Além disso, este estudo contribui para construção das definições operacionais que podem ser utilizados em estudos de intervenção, o que é algo relevante, devido à sua importância crescente como determinante para a qualidade de vida e bem-estar da pessoa idosa.

## Data Availability

Os dados de pesquisa estão disponíveis no corpo do artigo.
